# 

**Published:** 1890-03-29

**Authors:** 


### NEW OFFICES IN THE STRAND.

The Hospital *is now published at* 140, Strand, W.C. *It has been found desirable to establish* The Hospital *in this central and convenient locality, owing to the rapid extension of the business of this paper of late.*

### NOTICE TO CORRESPONDENTS.

All MS., Letters, Books for Review, and other matters intended for the Editor should be addressed The Editor, The Lodge, Porchester Square, London, W.

**Notice to Advertisers and Subscribers.**

All Advertisements, Orders for Copies of The Hospital, and Business Communications should be addressed to The Publisher, *not to the Editor*, The Hospital, 140, Strand, W.C.

**Bound Volumes of "The Hospital."**

Vol. VI., containing full page portraits of T.R.H. the Prince and Princess of Wales, and Miss Florence Nightingale, is now ready, 4s. post free. Cases for binding may be had of the Publisher, at 1s. 6d. each.

**To Residents, Secretaries, and Matrons.**

The Editor will be glad to have the Regulations and each Report as issued by Asylums, Hospitals, Convalescent and Nursing Institutions, as well as those of other Charities, and an early copy in duplicate of all Appeals and Festival Notices, etc., addressed to *The Editor, The Lodge, Porchester Square, London, W.* Any superintendent, secretary, matron, or other chief official who regularly sends such information may be registered, on application to the Publisher, to receive free of cost a cover for binding The Hospital in half-yearly volumes.

### Scraps and Gleanings.

Influenza is very bad in Cyprus.

Yellow fever has broken out in Brazil.

A leper asylum is being built at Gatomba, in Japan.

Mrs. Shaw is forming an ambulance class at Cambridge.

New York proposes to adopt a muzzling Act, as hydrophobia is on the increase.

A bazaar is being organised in aid of the Glasgow Samaritan Hospital for Women.

Bexley Cottage Hospital treated 53 in-patients last year; funds are satisfactory.

The Medical staff of Suffolk General Hospital have withdrawn their resignations.

Mr. J. J. Pridie, M.B., has been appointed house surgeon to Southport Infirmary.

Lord Cadogan has given a valuable bit of ground at Chelsea to the Guinness Trust.

The will of the late Sir W. Gull has been proved, and the personalty valued at £344,000.

St. Pancras Infirmary is to have outside iron stairs constructed in case of fire at a cost of £815.

Suffolk General Hospital treated 485 in-patients last year. The total expenditure was £2,925.

The Empress Frederick lately went over the Children's Hospital at Berlin with Professor Virchow.

The Grosvenor Hospital for Women and Children is appealing for £14,000 to provide new buildings.

Richmond Hospital issues its twenty-second annual report, which is most satisfactory in every respect.

Mr. W. G. Boase, M.R.C.S., has been appointed assistant house surgeon to the Rotherham Hospital.

Dublin medical students amused themselves by rioting last Saturday; eight were arrested, and fined £1 each.

At the Festival Dinner of the Middlesex Hospital donations to the amount of £2,700 were acknowledged.

The Chorley Dispensary treated 516 patients last year; amongst these there were seven deaths from consumption.

The Royal Chest Hospital in the City-road issues a special appeal for funds, signed by Lord Charles Bruce, and Mr. S. Hope Morley.

Mr. Cadbury is conferring with seven other gentlemen on the best means of using Moseley Hall as a sanatorium for women and children.

Woodlands Convalescent Home, near Bradford, is open winter and summer; it sometimes has 20 patients, sometimes 100 within its walls.

The Brabazon Home of Comfort for Members of the Girls' Friendly Society is a very useful convalescent and invalid institution at Reigate.

Silloth Convalescent Institution for Cumberland and Westmoreland treated 566 patients last year at an average cost of £1 3s. 8½d. per patient.

Finsbury Dispensary.—The report for 1889 shows that there have been 18,000 new cases, and 42,468 attendances of patients at the dispensary.

The National Truss Society issues a full and excellent report of its last year's work, when 2,386 persons were supplied with surgical appliances.

On Sunday afternoon, March 30th, at four p.m., the Rev. E. C. Hawkins will preach at St. Philip's, Regent-street, in aid of the East London Nursing Society.

The library of the Obstetrical Society has been removed from 54, Berners Street, W., to 20, Hanover Square, W., where all communications should be addressed.

The Guild of St. Paul, Cannes, the secretary of which is Miss Ward, has kindly sent a handsome present of flowers for the sick sailors in the Seamen's Hospital at Greenwich.

Meanwood Convalescent Home.—The cost of the 245 children received in the Home during the year was £364 13s. 6d., or £1 9s. 9½d. per head for the term of three weeks.

The Royal Hospital for Diseases of the Chest issues its 76th annual report. Over 400 in, and 5,000 out-patients were treated. Stock has had to be sold out to make up deficiency in income.

Lady Hillingdon has opened a Convalescent Home for Children at Lord Hillingdon's place in Kent. It is intended for patients from London. A sister from one of the metropolitan hospitals has been appointed matron.

The number of deaths from typhoid fever in Melbourne and suburbs during the year 1889 was 558, as against 240 in 1888, and 338 in 1887. The number of deaths from diphtheria in 1889 was 329, as against 130 in 1888, and 64 in 1887.

The Royal Free Hospital having boasted it had reduced its cost per bed for alcohol from £5 to £2, the secretary of St. Mary's wrote to say that at that hospital they had reduced their cost for alcohol to £1 18s. per bed.

Mr. Thomas Hope, of New York, a native of Langholm, who died a few days ago, has left the residue of his estate to found a hospital at Langholm, for the benefit of aged and infirm persons belonging to the district. The amount is estimated at 400,000 dols.

Lockhart Hospital.—The daily average number of patients during the year has been 9, for an average of 29 days each. The hospital this year has in hand a balance of £125 3s. 10d., which includes a balance of £85 14s. 5d., brought forward from the last account.

Samaritan Society, St. Thomas's Hospital.—The report states that the number of annual subscribers is increasing, and that the donations and other subscriptions, which fell off in 1888 to the extent of £80, regained that amount in 1889. The money boxes in chapel, wards, &c., were less successful by £24.

